# Exploring the Impact of Safewards on Aggression and Coercion in Psychiatric Inpatient Care: Findings From a Swedish Longitudinal Quasi‐Experimental Trial

**DOI:** 10.1111/inm.70228

**Published:** 2026-02-02

**Authors:** Jenny Karlsson, Anna Björkdahl, Nina Gårevik, Lars Kjellin, Veikko Pelto‐Piri, Naimi Johansson

**Affiliations:** ^1^ Department of Clinical Neuroscience Centre for Psychiatry Research, Karolinska Institutet and Stockholm Health Care Services Stockholm Sweden; ^2^ Red Cross University College Stockholm Sweden; ^3^ Faculty of Medicine and Health University Health Care Research Center, Örebro University Örebro Sweden; ^4^ Department of Clinical Science and Education Karolinska Institutet Stockholm Sweden

**Keywords:** aggression, coercion, involuntary treatment, longitudinal study, mental disorders

## Abstract

Coercive measures in psychiatric inpatient care remain controversial and are often associated with negative experiences for both patients and staff. The Safewards model aims to reduce conflict and containment by fostering a safer and more therapeutic ward environment. However, evidence regarding its effectiveness is mixed. This study investigated the implementation and impact of Safewards in nine Swedish psychiatric inpatient wards, focusing on coercive measures, aggressive incidents, and the normalisation of the intervention. A quasi‐experimental, longitudinal design with comparison wards was used. Data were collected through administrative records on coercive measures, staff surveys for incident reports (SOAS‐R), and normalisation (S‐NoMAD). Mixed model regression analyses assessed changes over time in coercive measures. Wards implemented between two and five Safewards interventions. No statistically significant reductions were found in coercive measures or aggressive incidents. Although the effects on mechanical restraint were not statistically significant, the significant increase in normalisation and the declining trend in mechanical restraint suggest a potential shift. Partial implementation and contextual challenges likely restricted the model's full impact. The study was reported according to the TREND checklist.

## 1 Introduction

In psychiatric inpatient care, individuals who have experienced or witnessed coercive measures often describe it as traumatic, leading to feelings of being treated inhumanely, a loss of control, and increased distress (Brophy et al. 2016; Verbeke et al. 2019). Such experiences are associated with risks for trauma and re‐traumatization for patients and staff (Cusack et al. 2018). Research highlights that violent incidents in psychiatric inpatient care are influenced by environmental conditions, ward culture, and staff behaviour, as well as by patient behaviour (Bowers 2014). Additionally, many patients perceive psychiatric wards as stressful and unsafe. Contributing factors include locked doors, limited access to private spaces, and the potential use of coercive measures (Weltens et al. 2021). Considering the growing call for a paradigm shift toward psychiatric care that is more person‐centred, recovery‐oriented, and grounded in human rights (WHO and Office of the High Commissioner for Human Rights 2023), there is an urgent need to reduce the use of coercive measures.

In recent decades, several initiatives have been launched in Sweden to reduce conflict and containment in psychiatric inpatient care. Despite these efforts, national registers indicate that coercive measures have not decreased (Socialstyrelsen Swedish National Board of Health and Welfare 2023). Moreover, a recent report from the Swedish Health and Social Care Inspectorate (Inspektionen för vård och omsorg [IVO] 2025) identified serious systemic deficiencies in compulsory psychiatric care, including the use of coercive measures without legal justification, poor documentation, and a lack of patient rights protection.

An evidence‐based approach that has shown promise in addressing such challenges is the Safewards model, developed to promote a safer and more therapeutic ward environment by proactively reducing conflict (e.g., aggression, self‐harm, absconding) and containment (e.g., seclusion, mechanical restraint, forced medication) (Bowers et al. 2014). Safewards was developed following years of research and identifies six interrelated domains that together influence conflict and containment: staff structure, physical environment, hospital surroundings, patient community, patient characteristics, and regulatory frameworks. The Safewards model introduces ten interventions designed to foster a safer and more supportive ward environment by improving relationships and communication among patients and staff, as well as within each group (Table 1). These interventions aim to reduce potential triggers of conflict and strengthen a shared sense of safety and collaboration. A core principle is the active involvement of both staff and patients in shaping a positive and safe ward culture (Bowers 2014).

**TABLE 1:** The 10 Safewards interventions (Fletcher et al. 2019).

Intervention	Description
Clear mutual expectations	Patients and staff work together to create mutually agreed aspirations that apply to both groups equally
Soft words	Staff take great care with their tone and use of collaborative language. Staff reduce the limits faced by patients, create flexible options and use respect if limit setting is unavoidable
Talk down tips	De‐escalation process focuses on clarifying issues and finding solutions together. Staff maintain self‐control, respect and empathy
Positive words	Staff say something positive in handover about each patient. Staff use psychological explanations to describe challenging actions
Bad news mitigation	Staff understand, proactively plan for and mitigate the effects of bad news received by patients
Know each other	Patients and staff share some personal interest and ideas with each other, displayed in unit common areas
Mutual help meetings	Patients offer and receive mutual help and support through a daily, shared meeting
Calm down methods	Staff support patients to draw on their strengths and use/learn copings skills before the use of PRN medication or containment.
Reassurance	Staff touch base with every patient after every conflict on the unit and debrief as required
Discharge messages	Before discharge, patients leave messages of hope for other patients on a display in the unit

Several studies have assessed the impact of Safewards in reducing both conflict and containment across various mental health care settings. The original study, a cluster randomised controlled trial conducted in the UK involving 15 hospitals and 31 psychiatric wards (16 intervention, 15 control), demonstrated a 15% reduction in conflict events and a 26% decrease in the use of containment measures compared to control groups (Bowers et al. 2015). More recently, systematic reviews have synthesised findings from multiple studies examining the implementation and outcomes of the Safewards model across diverse mental health care settings. These reviews show mixed results regarding its impact on reducing conflict and containment (Mullen et al. 2022; Finch et al. 2022; Ward‐Stockham et al. 2024). While some studies reported statistically significant reductions in conflict and containment, others found no notable effects. Additionally, qualitative findings highlighted improvements in the ward atmosphere, communication and a greater sense of safety on the wards. No previous study has evaluated the staff normalisation process during Safewards implementation, that is, how staff gradually integrate the model into their everyday routines and interactions over time (May et al. 2009).

While traditional Safewards fidelity measures capture whether specific interventions are delivered as intended at a given point in time, they do not fully reflect the extent to which these practices become part of the ward's habitual way of working. Assessing normalisation therefore adds a complementary perspective since it indicates the depth and durability of behavioural and cultural integration among staff and can reveal long term implementation patterns that fidelity checks alone may overlook. Differences in study designs, outcome measures, populations, implementation and follow‐up periods, as well as the absence of control groups, make it difficult to establish a clear relationship between Safewards and reduced conflict and containment (Baumgardt et al. 2019; Bowers et al. 2015; Davies et al. 2020; Fletcher et al. 2017; Riding 2016; Stensgaard et al. 2018). These mixed findings underscore the importance of continued research with solid research design to estimate effects on coercive measures in the long term, comparing changes with a control group.

The primary aim of this study was to evaluate the effect of Safewards on the use of coercive measures. Secondary aims were to examine whether the implementation influenced the frequency or severity of aggressive incidents, and to explore how staff assessed the normalisation of Safewards into routine practice.

## 2 Methods

### 2.1 Study Design

This study employed a naturalistic longitudinal, quasi‐experimental design with comparison wards, with start of Safewards implementation in 2021 and was followed for up to 2 years. Data was gathered through administrative sources and surveys, including repeated measurements of use of coercive measures, aggression incidents, fidelity to the Safewards model and staff perceptions of normalisation related to integrating Safewards interventions into daily practice. The study was reported according to the TREND checklist.

### 2.2 Study Setting

In Sweden, compulsory psychiatric care is legally permitted only within the publicly funded system, in accordance with the law on Compulsory Psychiatric Care (Socialdepartementet 1991:1128). The use of coercion is strictly regulated and requires formal documentation. The legally regulated coercive measures include mechanical restraint, whereby patients are fastened to a bed using straps; treatment without consent (e.g., forced medication) and seclusion, where patients are isolated in a secluded area under staff supervision. Physical holding is permitted only for short durations to facilitate the use of other coercive measures and is not formally regulated under coercive legislation.

This study was conducted in general psychiatric inpatient wards representing both urban and semi‐urban/rural areas. It involved nine intervention wards across two Swedish regions, referred to as Clinics A (urban) and B (semi‐urban/rural). At the time of recruitment, Clinic A had no prior experience with Safewards, whereas Clinic B had recently made an official decision to begin implementing Safewards. A convenience sampling of participating wards at Clinic A and B was conducted in collaboration with the clinic directors, resulting in four participating wards from Clinic A and five from Clinic B.

Further, nine comparison wards were identified: four wards from other clinics at the same metropolitan region as Clinic A, hereafter referred to as Clinic C, and five wards from a comparable semi‐urban/rural region as Clinic B, hereafter referred to as Clinic D, where standard care was provided during the study period. The comparison wards were selected to match the intervention wards in terms of service type and population. Due to practical and limited resources, randomisation between wards was not feasible. Comparison wards were identified post‐implementation to strengthen the analysis of the primary outcome of coercive measures only and remained unaware of their role as comparisons, minimising the risk of behavioural changes due to study participation.

#### 2.2.1 Training and Implementation

Ward managers were asked to nominate two to three Safewards champions from their nursing staff to lead the implementation process. At both Clinic A and B, ward managers and nursing staff participated in separate 4‐h introductory workshops tailored to their respective clinics. These sessions were led by AB and VP from the research team and included an overview of the Safewards model background and detailed explanations of the 10 interventions, using materials from the Safewards website (Safewards.net). During the workshops, staff were divided into small groups to discuss and prioritise which interventions to implement first. A voting process determined the initial three to five interventions for each ward, with the expectation that these would be fully implemented within 1 year. Ward managers and designated champions were informed that they could request implementation support from the research team to discuss potential barriers or ideas.

### 2.3 Data Collection

#### 2.3.1 Administrative Data

Ward‐level information on coercive measures and bed days production was obtained from hospital administrative databases. Longitudinal monthly ward‐level data were collected from 2020 to 2022 for both intervention and comparison wards. The collected variables included the monthly number of bed days, involuntary bed days, and the use of coercive measures. Data on coercive measures were defined as the three types: (i) seclusion (up to 8 h), (ii) forced medication and (iii) mechanical restraint (up to 4 h). To account for potential bias introduced by individuals with exceptionally high exposure to coercive measures, an ‘outlier’ was defined as a patient with at least five coercive measures and accounting for more than 20% of all coercive measures on a given ward in a single year. The definition was informed by internal clinical discussions and patient‐level data from 2017 to 2019 retrieved from Clinics A and B and aimed to reduce the influence of atypical cases by chance on ward‐level trends.

#### 2.3.2 Survey Data

Incidents of patient aggression were assessed using the Staff Observation Aggression Scale–Revised (SOAS‐R), a standardised tool for systematically documenting aggressive incidents, including threats and violence, in psychiatric care settings (Nijman et al. 2005). The SOAS‐R measured both the frequency and severity of reported aggressive incidents. The severity was rated based on predefined criteria, including the trigger for aggression, type of aggression, the target, the consequences, and how the incident was resolved (Nijman et al. 1999). Each SOAS‐R data collection period lasted a month, during which nursing staff at the ward used the SOAS‐R checklist to document each observed aggressive incident, its date, time, characteristics and consequences. Three SOAS‐R data collections were conducted with 6 months intervals, with the first occasion held before the start of Safewards implementation.

The feasibility of the intervention and the extent to which Safewards was integrated and normalised in the care environment were evaluated using the Swedish version of the Normalisation Process Theory Measure, S‐NoMAD questionnaire, which captures staff perceptions of the intervention's integration into daily practice (Elf et al. 2018). The NoMAD instrument is retrievable from Finch et al. (2018). NoMAD is based on the Normalisation Process Theory (NPT) which explains how new work practices gradually become familiar and eventually fully integrated into everyday routines (May et al. 2009). NPT proposes that this integration occurs through four key constructs: coherence, cognitive participation, collective action, and reflexive monitoring, which together describe how individuals and teams make sense of, engage with, enact, and appraise a new practice. The questionnaire included 20 items rated on a five‐point Likert scale (from 1 = strongly disagree to 5 = strongly agree). The survey was scheduled to be completed quarterly by ward staff from the start of implementation and for 1 year.

A fidelity assessment was conducted to evaluate whether Safewards was implemented as intended. Intervention fidelity was collected using an adapted simplified version of the original Safewards Fidelity Checklist, a standardised tool available on the Safewards website (Safewards, n.d.). The checklist was completed by the research team in ward observation walk‐throughs, planned every 3 months throughout the study period.

### 2.4 Ethical Considerations

Ethical approval was obtained from the national Ethical Review Authority (EPM 2020–03881). All data were pseudonymized, with no personal identifiers collected. Ward level data were extracted from medical records and analysed only at group level, in accordance with ethical approval and Swedish regulations for the use of clinical registers; individual patient consent was not required. SOAS‐R and S‐NoMAD included staff signatures and were assigned serial numbers. Staff who agreed to participate gave their signed informed consent and responded anonymously.

### 2.5 Statistical Analysis

#### 2.5.1 Regression Analysis of Primary Outcome

To analyse the effect of implementation of Safewards on coercive measures, we modelled changes over time for intervention wards as compared to changes over time for comparison wards, in a mixed model regression analysis. This method enables comparison of changes over time between intervention and comparison wards, while accounting for repeated measurements within wards. Specifically, we applied a generalised linear mixed model with Poisson distribution and log link. The dependent variable was defined as the number of coercive measures (in counts), and the number of bed days used as offset term (the natural logarithm of bed days) to account for exposure. The Poisson distribution is characterised by a discrete, non‐negative function with variance equal to the expected mean and often used to model count data (Vittinghoff et al. 2012).

For the analysis three periods were defined: pre‐implementation period for the 12 months before introducing Safewards, Implementation period for the first 12 months of Safewards, and post‐implementation period for 13–24 months of follow‐up. Indicators for ward grouping (the comparison group as reference) and for the three periods (Pre‐period as reference), as well as the interaction between them, were included in the regression model as fixed effects. The interaction terms were used to estimate whether changes in coercive measures over time differed between wards that implemented Safewards and those that did not. Further, time was centred around the month of implementation for the intervention wards (i.e., month since the start of Safewards in early 2021). For comparison wards, time was centred around January 2021. Time measured in months since implementation was included as a continuous (linear) fixed effect. The regression model included random intercepts and random slopes of each ward to account for the interdependence between observations from the same ward. The random effects were modelled with an unstructured covariance.

The results are presented as incident rate ratios (IRRs), that is, how many times more or less common the incidents were comparing the groups, with 95% confidence intervals. The analysis was conducted for coercive measures overall, and for the three subcategories mechanical restraint, seclusion, and forced medication. To test the robustness of the results, we conducted sensitivity analyses by excluding outliers and by using involuntary bed days as offset term. This data was only available for subsets of the wards. Specifically, the number of coercive measures after excluding outliers was available for wards in Clinic A, B and C, and the number of involuntary bed days was available for wards in Clinic A, C and D. Statistical analyses were conducted using IBM SPSS Statistics and Stata/MP 18.0.

#### 2.5.2 Analysis of Secondary Outcomes

The survey data of aggressive incidents, normalisation and fidelity were presented to show trends over time with descriptive statistics by count, mean, median, proportion, standard error and 95% confidence intervals as appropriate. For SOAS‐R, the number of aggression incidents for each ward was calculated, and the survey items were summed to an aggression severity score of each incident with a maximum score of 22. For the S‐NoMAD survey, an overall normalisation score was defined as the mean of the 20 S‐NoMAD items for each responding staff member (Finch et al. 2018). For the Fidelity Checklist, a fidelity score for each ward was defined as the proportion of Safewards interventions implemented as intended (e.g., three interventions visibly implemented out of five interventions reported as introduced). A score of 100% indicated full implementation of the interventions a ward claimed to have introduced. To analyse trends over time, differences in aggression and normalisation scores between first and second, and between first and third data collection were tested using a Mann–Whitney test. The ward‐specific fidelity score was not assessed in statistical testing due to the limited number of ward observations.

## 3 Results

### 3.1 Descriptive Statistics

Table 2 presents background characteristics of the intervention wards. The wards ranged in size from 12 to 40 beds, and the proportion of involuntary to voluntary bed days varied across wards. Data on involuntary bed days and physician staffing from Clinic B, as well as patient age distribution from Clinic A, were missing despite repeated requests for administrative data. Staffing levels varied, with the number of full‐time equivalent (FTE) nurses per bed ranging from 0.29 to 1.33, and nursing assistants from 1.00 to 2.00 per bed. The proportion of female patients ranged from 32% to 68%, and the median patient age was approximately 40 years. Both intervention and comparison wards represented various psychiatric specialisations such as psychotic disorders, affective disorders, or general adult psychiatry. Ward B25 was combined with another (non‐Safewards) ward and quit implementation of Safewards in July 2021. Hence, no further survey data was collected, and Ward B25 was included in the analyses only until July 2021.

**TABLE 2:** Descriptive data of intervention wards, year 2022.

Ward	A11	A12	A13	A14	B21	B22	B23	B24	B25^a^
No. of beds	40	21	16	16	14	14	12	12	20
No. of bed days, annual	12 789	5789	5103	5251	5504	4359	3121	4987	6520
… of which involuntary bed days	7538	1458	2008	3591	—	—	—	—	—
No. of Safewards interventions implemented	4	3	5	4	3	3	3	2	0
**Staff, no. per bed** ^b^									
Physicians	0.10	0.09	0.09	0.09	—	—	—	—	—
Nurses	0.56	0.70	0.53	0.53	0.93	0.29	0.33	1.33	1.05
Nursing assistants	1.39	1.22	1.05	1.05	1.29	1.00	1.08	1.75	2.00
Other staff	0.08	0.04	0.01	0.01	0.43	0.29	0.08	1.00	0.60
**Patient characteristics**									
Age, median	—	—	—	—	40	41	43	39	38
Women (%)	38	68	57	41	49	62	62	32	52
**ICD‐10 diagnose groups** ^**c**^ **(%)**									
F00–F09	0	1	0	0	0	4	4	1	5
F10–F19	8	4	4	5	6	6	6	81	17
F20–F29	60	8	6	58	91	21	16	12	22
F30–F39	5	27	45	5	4	46	49	11	39
F40–F48	9	35	23	6	11	55	64	21	39
F50–F59	0	2	0	0	1	4	1	1	1
F60–F69	2	7	2	3	4	12	7	6	10
F70–F79	1	1	1	0	4	8	8	2	4
F80–F89	3	2	3	3	7	16	14	4	9
F90–F98	0	2	2	2	7	13	17	12	11
Z00–Z99	9	8	6	14	3	4	8	33	11
Other diagnose groups	3	3	8	4	7	14	19	10	21

^a^ Ward B 25 was combined with another ward and quit implementation of Safewards in July 2021. Therefore, no survey data was collected, and the ward was included in the analyses of coercive interventions only up until July 2021. The annual number of bed days for this ward refers to 2021.

^b^ Staff, number per bed, refers to the number of full‐time equivalents of physicians, nurses, and nursing assistants per bed.

^c^ Diagnose groups (ICD‐10‐SE) F00–F09: Organic, including symptomatic, mental disorders. F10–F19: Mental and behavioural disorders due to psychoactive substances. F20–F29: Schizophrenia, schizotypal, and delusional disorders. F30–F39: Mood (affective) disorders. F40–F48: Neurotic, stress‐related, and somatoform disorders. F50–F59: Behavioural syndromes associated with physiological disturbances and physical factors. F60–F69: Disorders of adult personality and behaviour. F70–F79: Intellectual disabilities. F80–F89: Disorders of psychological development. F90–F98: Behavioural and emotional disorders with onset usually occurring in childhood and adolescence. Z00–Z99: Factors influencing health status and contact with health services. In Clinic B, all patients' diagnoses were reported, hence the percentages sum to more than 100%.

**FIGURE 1:**
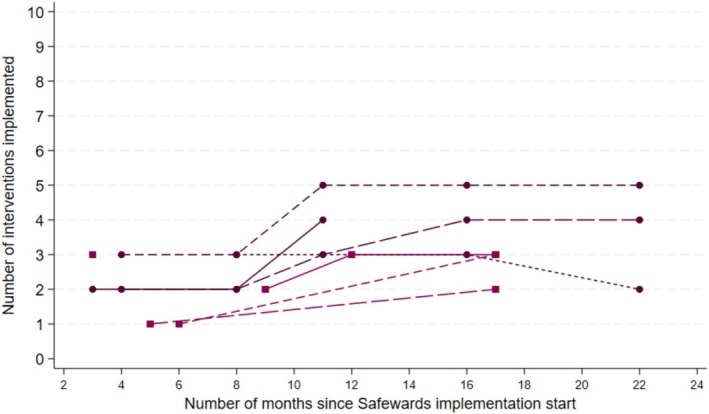
Number of Safewards interventions implemented at each ward. Each line in the graph represents a ward, with points indicating the time of data collection. Months refer to the month of data collection the interventions were implemented by this month or earlier.

**FIGURE 2:**
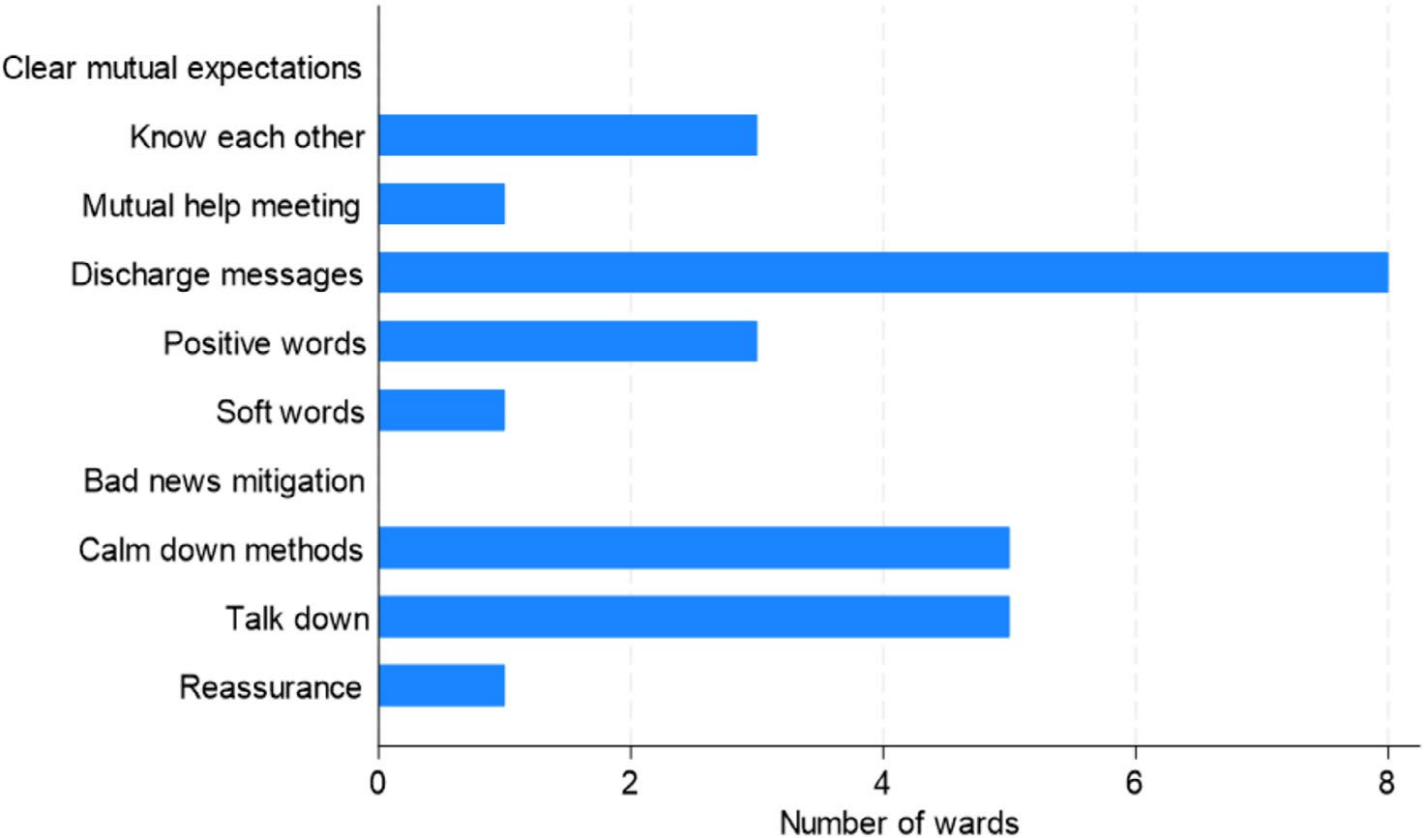
Number of wards that have implemented each of the Safewards interventions.

Figure 1 presents the number of Safewards interventions implemented at each ward over time. One ward implemented five interventions, two wards implemented four interventions, and the remaining wards implemented two or three interventions. ‘*Discharge messages*’ was the most common intervention implemented at eight of the wards; the second most common was ‘Calm down methods’ and ‘*Talk down*’ at five of the wards (Figure 2). None of the wards implemented *Clear Mutual Expectations* or *Bad News Mitigation*.

### 3.2 Primary Outcome: Coercive Measures

**FIGURE 3:**
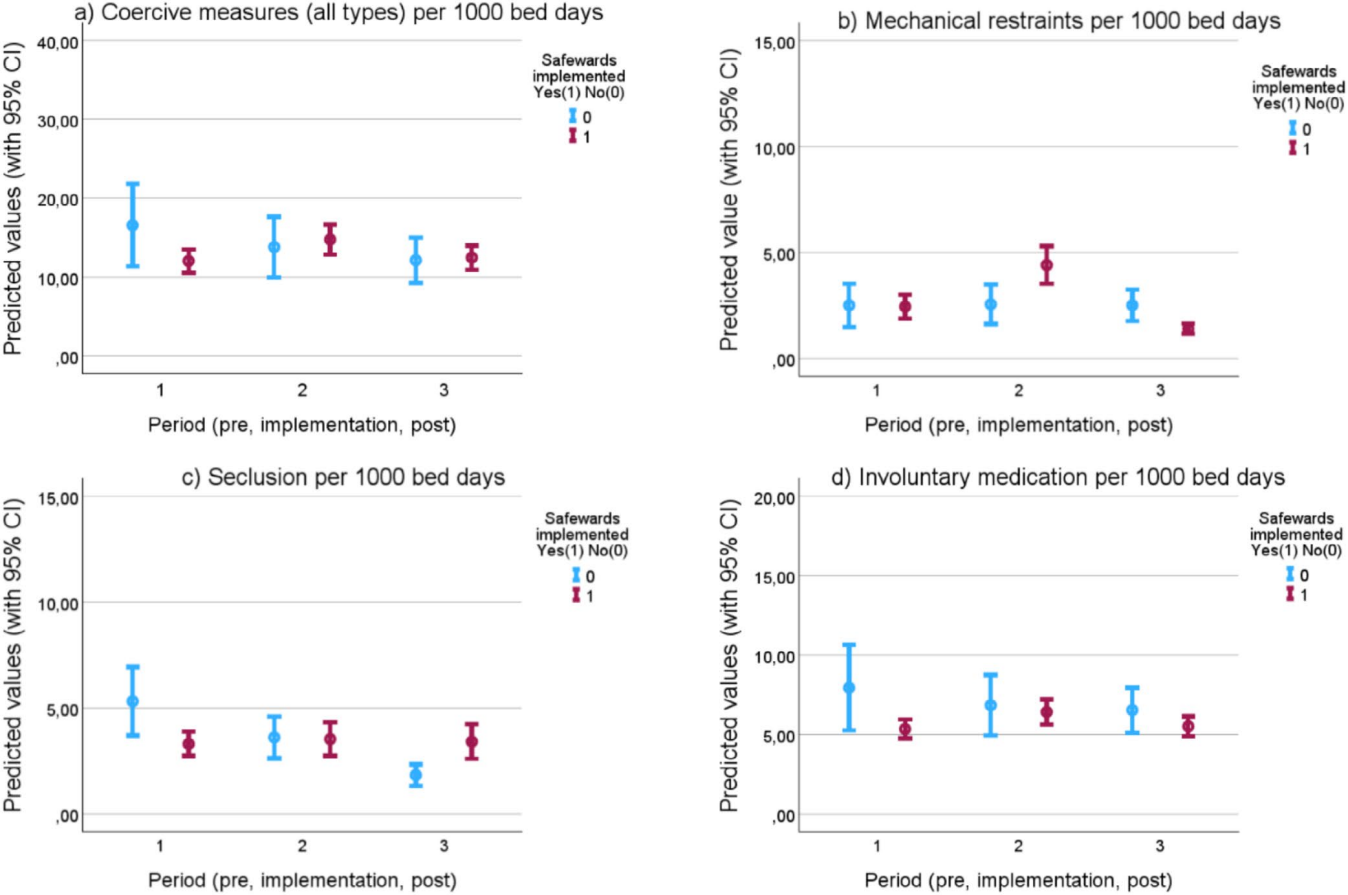
Predicted values of coercive measures, by type. Each point in the graph represents the estimated predicted number of (a) coercive measures all types, (b) mechanical restraint, (c) seclusion, (d) forced medication, across all wards over twelve months in the given period. The bars show a 95% confidence interval. Pre‐implementation period (1) refers to the 12 months before Safewards implementation, implementation period (2) to the first 12 months of implementation, and post‐implementation period (3) to months 13–24 after implementation start.

Unadjusted trends in coercive measures remained stable over the study period, with a monthly mean ranging between 15 and 20 coercive measures per 1000 bed days in both intervention and comparison wards (Appendix S1: Figures A1–A4). In regression analyses, we estimated changes in coercive measures from the pre‐implementation period to the implementation and post‐implementation periods, comparing intervention wards to the changes in comparison wards over the same timeframes. The incidence rate ratio (IRR) for coercive measures was 1.11 (95% CI 0.73–1.67) during the implementation period and 0.69 (95% CI 0.37–1.29) in the post‐implementation period (Table 3). The point estimates suggest a non‐significant increase in coercive measures during implementation, followed by a non‐significant decrease post‐implementation. The results are presented graphically in Figure 3, showing the predicted number of coercive measures per 1000 bed days.

**TABLE 3:** Incidence rate ratio of coercive measure, estimated in regression analysis.

	Incidence rate ratio	95% confidence interval	*p*
**Coercive measures**			
Implementation period^a^	1.109	0.734–1.674	0.623
Post‐implementation	0.692	0.372–1.289	0.246
No. of observations	607		
No. of wards	18		
**Mechanical restraint**			
Implementation period	1.459	0.726–2.931	0.288
Post‐implementation	0.351	0.117–1.050	0.061
No. of observations	607		
No. of wards	18		
**Seclusion**			
Implementation period	0.813	0.419–1.580	0.541
Post‐implementation	0.628	0.201–1.965	0.423
No. of observations	607		
No. of wards	18		
**Forced medication**			
Implementation period	1.022	0.674–1.548	0.920
Post‐implementation	0.622	0.325–1.187	0.149
No. of observations	607		
No. of wards	18		
**Sensitivity analyses**			
*Coercive measures excl. Outliers* ^b^			
Implementation period	1.466	0.888–2.420	0.134
Post‐implementation	1.031	0.505–2.103	0.933
No. of observations	427		
No. of wards	13		
*Coercive measures per involuntary bed days* ^c^			
Implementation period	1.073	0.568–2.024	0.828
Post‐implementation	0.447	0.159–1.258	0.127
No. of observations	464		
No. of wards	13		

^a^ Pre‐implementation period as the reference (IRR 1.000), referring to the 12 months before Safewards implementation. Implementation period refers to the first 12 months of implementation, and post‐implementation to months 13–24 after implementation start.

^b^ Sensitivity analyses include a limited number of wards, due to lack of data: Coercive interventions excl outliers include nine intervention wards and four comparison wards (Clinic A, B and C).

^c^ Sensitivity analyses include a limited number of wards, due to lack of data: Coercive interventions per involuntary bed day include four intervention wards and nine comparison wards (Clinic A, C, and D).

The results were similar when subcategorizing by type of coercive measure (seclusion, forced medication, mechanical restraints), with one exception: a more pronounced decrease in the post‐implementation period for mechanical restraints, with an IRR of 0.35 (95% CI: 0.12–1.05) and a *p*‐value of 0.061. Sensitivity analyses confirmed the main findings—the results remained stable and statistically insignificant when excluding coercive measures of patient ‘outliers’, and when coercive measures were analysed per involuntary bed days.

### 3.3 Secondary Outcomes

Aggressive incidents, measured using the SOAS‐R, were completed by all nine intervention wards prior to the implementation of Safewards, with a total of 146 aggressive incidents reported and a mean of 16.11 (standard error 4.37) incidents per ward (Table 4). Five wards completed the following data collections, with a mean of 20.60 (SE 5.56) incidents per ward during the implementation period, and 8.80 (SE 2.04) during the post‐implementation period. The mean (SE) aggression severity score was 7.10 (0.36) in the pre‐period, 7.50 (0.45) in the implementation period and 8.23 (0.68) in the post‐period. However, there was no statistically significant difference in aggression scores between the pre‐ and implementation periods (*p* = 0.458) or between the pre‐ and post‐implementation periods (*p* = 0.093) at the 5% significance level.

**TABLE 4:** Results of number and severity score of aggressive incidents (SOAS‐R).

Data collection	Pre‐period	Implementation‐ period	Post‐period
No. of wards^a^	9	5	5
Months^b^, median (min; max)	−5 (−14; −4)	8 (3; 10)	22 (19; 22)
Total no. of incidents^c^	146	102	44
No. of incidents^d^ Mean (SE) (95% CI)	16.111 (4.370) (6.034; 26.188)	20.600 (5.564) (5.151; 36.049)	8.800 (2.035) (3.151; 14.449)
Severity score^e^ Mean (SE) (95% CI)	7.096 (0.363) (6.379; 7.812)	7.500 (0.452) (6.603; 8.397)	8.227 (0.683) (6.849; 9.606)
*p* ^f^	—	0.458	0.093

^a^ Number of wards completing data collection.

^b^ Timing of data collection, in months since Safewards intervention started.

^c^ Total number of incidents (all wards).

^d^ Mean number of aggressive incidents reported per ward.

^e^ Mean Aggressive incident severity score, across all incidents.

^f^ Aggressive incident severity score, across all incidents: *p* of Mann–Whitney test (Wilcoxon two‐sample rank‐sum test) with null hypothesis that the aggression score of the 2nd and 3rd data collection, respectively, follows the same distribution as the 1st data collection.

Normalisation of Safewards into everyday work, as measured by the S‐NoMAD survey, showed an increasing trend over time (Table 5). The mean score for staff perceptions of normalisation improved significantly between the first and second data collection points, as well as between the first and third (*p* < 0.05), indicating greater integration of the Safewards model into daily routines. Eight wards completed the S‐NoMAD survey at the first data collection, four at the second, and seven at the third, with the number of staff respondents ranging from 79 to 116. However, response rates declined in later rounds, with only three wards participating in the fourth data collection and two in the fifth. The Fidelity Checklist showed a similar pattern of missing data. Fidelity scores demonstrated a progressive increase over time, with several wards reaching full implementation of the selected interventions by the final evaluation.

**TABLE 5:** Results of normalisation score (S‐NoMAD) and implementation fidelity.

Data collection	1st	2nd	3rd	4th	5th
**S‐NoMAD**					
No. of wards^a^	8	4	7	3	2
Months^b^, median (min; max)	4.5 (3; 9)	8 (8; 12)	16 (11; 17)	16 (16; 22)	22 (22; 22)
No. of respondents^c^	116	79	104	36	30
Normalisation score^d^ Mean (SE) (95% CI)	3.686 (0.050) (3.586–3.786)	3.849 (0.069) (3.711–3.986)	3.858 (0.544) (3.750–3.966)	—	—
*p* ^e^	—	0.018	0.010	—	—
Two wards norm. score^f^ Mean (SE) (95% CI)	3.919 (0.083) (3.749–4.089)	3.927 (0.108) (3.707–4.417)	3.904 (0.105) (3.690–4.119)	4.065 (0.112) (3.836–4.295)	4.119 (0.077) (3.961–4.278)
**Fidelity**					
No. of wards^a^	5	3	6	4	2
Months^b^, median (min; max)	4.5 (3; 5)	8 (8; 12)	13.5 (11; 17)	19 (16; 22)	22 (22; 22)
Fidelity score^g^ (%)	0.733	0.889	0.889	0.938	1.000

^a^ Number of wards completing data collection.

^b^ Timing of data collection, in months since Safewards intervention started.

^c^ Total number of staff respondents (all wards).

^d^ Mean Normalisation score across all respondents.

^e^ Normalisation score, across all respondents: *p*‐value of Mann–Whitney test (Wilcoxon two‐sample rank‐sum test) with null hypothesis that the normalisation score of the 2nd and 3rd data collection, respectively, follows the same distribution as the 1st data collection.

^f^ Mean Normalisation score of respondents from the two wards that completed all five data collections.

^g^ Fidelity score percentage (all wards).

## 4 Discussion

This study primarily aimed to assess the impact of Safewards on the use of coercive measures, and secondarily to explore changes in aggression incidents and normalisation over time. No statistically significant reductions in coercive measures or aggression incidents were found, although a non‐significant downward trend in mechanical restraint was observed. Normalisation of the implementation of Safewards showed a significant increase over time. Results from previous research on Safewards are mixed, as highlighted in several recent reviews (e.g., Finch et al. 2022; Ward‐Stockham et al. 2022; Mullen et al. 2022).

Comparisons of our results are most relevant with studies conducted in general adult psychiatric inpatient care. Many such studies report positive effects, including reduced conflict and containment, and improved ward climate (e.g., Bowers et al. 2015; Stensgaard et al. 2018; Lickiewicz et al. 2021). Whereas studies in forensic settings often show no significant change, likely reflecting differences in patient profiles, security levels, and routines (Maguire et al. 2018; Price et al. 2016). Methodologically, many prior studies evaluating Safewards were conducted in one or two wards without a comparison group (e.g., Baumgardt et al. 2019; Lickiewicz et al. 2021; Maguire et al. 2018; Davies et al. 2020; Riding 2016) or have used simple pre‐post designs with only two measurement points (e.g., Baumgardt et al. 2019; Maguire et al. 2018; Davies et al. 2020). In contrast, this study collected repeated monthly measurements of coercive measures across a 3‐year period and included comparison wards. Moreover, previous studies have used a wide range of outcome measures, complicating comparisons. While some focused on coercive measures similar to this study (e.g., Stensgaard et al. 2018), others adopted broader constructs such as Bowers et al. (2015) using ‘conflict’ and ‘containment’ as primary outcomes, which included incidents such as absconding and self‐harm. These types of incidents were not available in our data; therefore we cannot assess whether such outcomes changed in our intervention wards.

In addition, previous studies varied in the extent to which they implemented the Safewards model. Most prior studies implemented the full model with all ten interventions (e.g., Bowers et al. 2015; Stensgaard et al. 2018; Fletcher et al. 2017). In contrast, our 24‐month naturalistic implementation resulted in the adoption of only two to five interventions per ward. The most used interventions were *Discharge Messages*, *Calm Down Methods*, and *Talk Down*, while none of the wards implemented *Clear Mutual Expectations* or *Bad News Mitigation*. Lickiewicz et al. (2021) demonstrated that targeted implementation of a limited number of interventions can still yield positive results. However, the partial implementation in the present study may help explain the lack of significant reductions in coercive measures, as the model's effectiveness may depend on the cumulative impact of all ten interventions.

Estimating a ‘null‐effect’ of the change in coercive measures across all intervention wards raises concerns of variation between wards. However, unadjusted trends in coercive measures (Appendix S1: Figures A2–A5) indicated stable patterns over time in both intervention and comparison wards. Some wards had consistently higher levels and greater variation, others had lower and more stable rates, only two wards demonstrated a clear negative trend over time. We also examined whether patient ‘outliers’ influenced the results, by excluding them in sensitivity analyses and the results remained unchanged. This suggests that the absence of a reduction cannot be explained by a few high‐frequency patients. Yet, cultural context likely influenced the results. Unlike Denmark and Australia, where national policies explicitly support the reduction of restrictive practices (Fletcher et al. 2017; Stensgaard et al. 2018), Sweden have national guidelines and recommendations, but they are general and do not specify required practices, staff competencies, or measurable reductions in coercive measures. This absence of clear directives and structured support from government and healthcare authorities may have contributed to fragmented implementation across wards. Addressing this gap through clear national policies or targets could support more consistent implementation and improve outcomes in Swedish psychiatric inpatient settings.

A longer implementation period may support the integration of new practices (May et al. 2009), consistent with our finding that normalisation, measured by the S‐NoMAD instrument, increased significantly over time. This suggests that staff gradually began to perceive Safewards as an integrated part of everyday practice, potentially indicating a cultural shift within the wards. Further, this highlights the value of assessing staff perceptions to understand how interventions become embedded, even when quantitative measures show little or no change. These findings align with known implementation success factors, such as sufficient resources, strong leadership, active involvement from staff and patients, ongoing support, and adequate time when implementing complex interventions like Safewards (Björkdahl et al. 2024; Fletcher et al. 2021; Knauf et al. 2023; Pelto‐Piri et al. 2024; Karlsson et al. 2025). Despite the lack of statistically significant reductions in coercive measures overall, the non‐significant downward trend in mechanical restraint may signal the beginning of a cultural shift. The absence of statistically significant effects may also reflect limited statistical power, variability in adherence, or contextual barriers, rather than true lack of impact.

While this study focused on quantitative data, they may not capture the full scope of Safewards' impact. Qualitative research often highlights positive effects, including improved ward atmosphere, increased patient involvement, and strengthened nursing practices (e.g., Finch et al. 2022; Kennedy et al. 2019; Mullen et al. 2022; Ward‐Stockham et al. 2022). These findings suggest that Safewards may contribute not only to reducing conflict and containment but also to fostering more therapeutic, inclusive, and person‐centred ward cultures. In this light, confidence in the model is grounded not only in measurable outcomes but also in its alignment with core values of psychiatric safety, partnership, and recovery‐oriented care. A mixed‐methods approach could have offered a more nuanced perspective, capturing cultural and relational changes that are not easily captured by statistics alone. The absence of statistically significant findings does not imply ineffectiveness but rather highlights the limitations of relying solely on quantitative measures when evaluating complex interventions. Taken together, the study provides limited support for Safewards' impact on coercive measures and aggressive incidents but indicates increased normalisation in the implementation of Safewards over time. These findings emphasise the need for long‐term, well‐supported implementations, and highlight the value of combining quantitative and qualitative data to fully capture the model's impact.

### 4.1 Methodological Considerations

The use of both administrative and staff‐reported survey data is a strength of this study, but data reliability must be considered. The administrative data on coercive measures may be inconsistent, as shortcomings in documentation and compliance have been reported (IVO 2025). Continuous reporting of aggressive incidents (SOAS‐R) may introduce ‘measurement fatigue’ where staff underreport milder incidents over time, which could explain the non‐significant upward trend in the severity of aggressive incidents (Nijman et al. 2005).

The NoMAD survey relies on staff self‐reports and focuses on individual and team‐level processes, which may not fully capture structural barriers such as staffing shortages or competing priorities (May et al. 2009). The Fidelity checklist primarily focuses on visible signs of Safewards rather than exploring its integration into everyday work, which may overlook contextual factors, staff engagement, and variations in implementation (Bowers et al. 2015; Baumgardt et al. 2019; Fletcher et al. 2019). In this study, fidelity was assessed using a simplified version of the original checklist, emphasising the observable presence of interventions over their incorporation into everyday work. In contrast to previous studies where fidelity reflected the number of all ten interventions visibly in place (e.g., Bowers et al. 2015; Fletcher et al. 2017; Dickens et al. 2020), we defined fidelity relative to the number of interventions each ward had introduced. This approach was chosen to better capture partial implementation in a real‐world context, acknowledging that full adoption of all ten interventions was rarely feasible in our setting. Although fidelity scores increased over time, this method makes it difficult to determine to what extent Safewards has been implemented as originally intended. Future research should therefore consider using complementary evaluation methods to better capture implementation fidelity beyond visible components.

Given the naturalistic study setting, the research team encountered difficulties in collecting survey data as planned. Missing data is a well‐documented challenge in longitudinal data collections (Little and Rubin 2019) and has also been noted in previous Safewards studies (e.g., Dickens et al. 2020). In addition, the implementation of Safewards began in early 2021, during the COVID‐19 pandemic when restrictions and changes in routines influenced both the implementation process and data collection. Due to varying response rates during data collection, the analyses could not be conducted exactly as originally planned. Instead, changes in normalisation and the occurrence of aggressive incidents were analysed using combined data from all wards at each point of measurement, rather than on a ward‐by‐ward basis.

### 4.2 Strengths and Limitations

One of the main strengths of this study is the repeated measurements for all outcome measures. Many previous Safewards studies had short implementation and/or follow‐up periods, for example 8–12 weeks (Bowers et al. 2015; Price et al. 2016). Even the longest earlier trial, the Victorian Safewards trial, covered 18 months including follow‐up (Fletcher et al. 2017). Our extended 24‐month period provides valuable insights into implementation in a naturalistic setting. In addition, the use of comparison wards strengthened our analysis.

This study also has several limitations. First, the lack of patient perspectives limits the understanding of how Safewards may have influenced the wards' environment. Some previous studies have incorporated patient perspectives using instruments such as the Developing Recovery Enhancing Environments Measure (DREEM) (Cabral and Carthy 2017, Dinniss et al. 2007). Similarly, the use of validated instruments such as EssenCES (e.g., for assessing social climate), the Ward Atmosphere Scale (Tomlin and Tonkin 2023), or the VPC‐14 (Hallett et al. 2018), which captures patients' perceptions of feeling valued, protected, and cared for, might have provided a more nuanced understanding of the Safewards impact from the service users' point of view. Second, regarding the comparison wards, although they did not implement Safewards, we lacked information on whether they used other strategies to reduce conflict and containment during the study period. Finally, as in most Safewards studies, data were analysed at ward level, resulting in small sample sizes and limited statistical power. Patient‐level data on coercion and aggression would increase power and enable more detailed analyses of influencing factors.

## 5 Conclusion

The results showed that implementation of the Safewards model did not lead to statistically significant reductions in coercive measures or aggressive incidents. However, a non‐significant downward trend in the use of mechanical restraints, combined with significantly increased normalisation, suggests that even partial implementation of the model may support cultural shifts within psychiatric inpatient care. The findings highlight the inherent challenges of implementing complex interventions in psychiatric settings and underscore the need for sustained support, contextual adaptation, and the integration of both quantitative and qualitative methods to fully capture the intervention's impact.

## 6 Relevance for Clinical Practice

The findings underscore that implementing complex interventions such as Safewards does not automatically lead to measurable reductions in coercion or aggression, even when the intervention is evidence‐based and supported internationally. For clinical practice, this highlights the necessity of moving beyond a ‘one‐size‐fits‐all’ approach. Before wide‐scale adoption, it is essential to assess the readiness of the clinical setting, ensure that sufficient time and resources are allocated, and provide ongoing staff training and support. Even partial implementation may bring subtle but meaningful cultural shifts, such as improved normalisation of Safewards related care, that are not immediately visible in quantitative outcome measures but can still enhance the therapeutic environment for both patients and staff.

## Author Contributions

The research idea was developed by A.B. and V.P.‐P. The study design was carried out by A.B., V.P.‐P., L.K., J.K., and N.J. Statistical analysis was conducted by N.J., and J.K. The manuscript was written by J.K., N.J., and N.G., with significant input and critical revisions from L.K., A.B., and V.P.‐P. All authors reviewed and approved the final version of the manuscript.

## Funding

This study was funded by the independent and non‐profit organisations AFA Insurance (D‐nr 190272) and FORTE (2021/00266).

## Ethics Statement

Ethical approval was obtained from the national Ethical Review Authority (EPM 2020–03881).

## Consent

Individual patient consent was not required. Ward level data were extracted from medical records and analysed at group level, in accordance with ethical approval and Swedish regulations for the use of clinical registers.

## Conflicts of Interest

The authors declare no conflicts of interest.

## Supporting information


**Appendix S1:** inm70228‐sup‐0001‐AppendixS1.docx.

## Data Availability

The data that support the findings of this study are available from the corresponding author upon reasonable request.
