# Supplementary material for: Exploring the Impact of Safewards on Aggression and Coercion in Psychiatric Inpatient Care: Findings From a Swedish Longitudinal Quasi‐Experimental Trial
Source: Int J Ment Health Nurs. 2026 Feb 2;35(1):e70228. doi: 10.1111/inm.70228 (PMC12865338; doi:10.1111/inm.70228)
Supplement: Supplementary file 1 — Appendix S1: inm70228‐sup‐0001‐AppendixS1.docx. [file INM-35-0-s001.docx]

**Exploring the impact of Safewards on aggression and coercion in psychiatric inpatient care: findings from a Swedish longitudinal quasi-experimental trial**

**APPENDIX**

Figure A1. Coercive measures per 1000 bed days, mean of Safewards wards and control wards, respectively


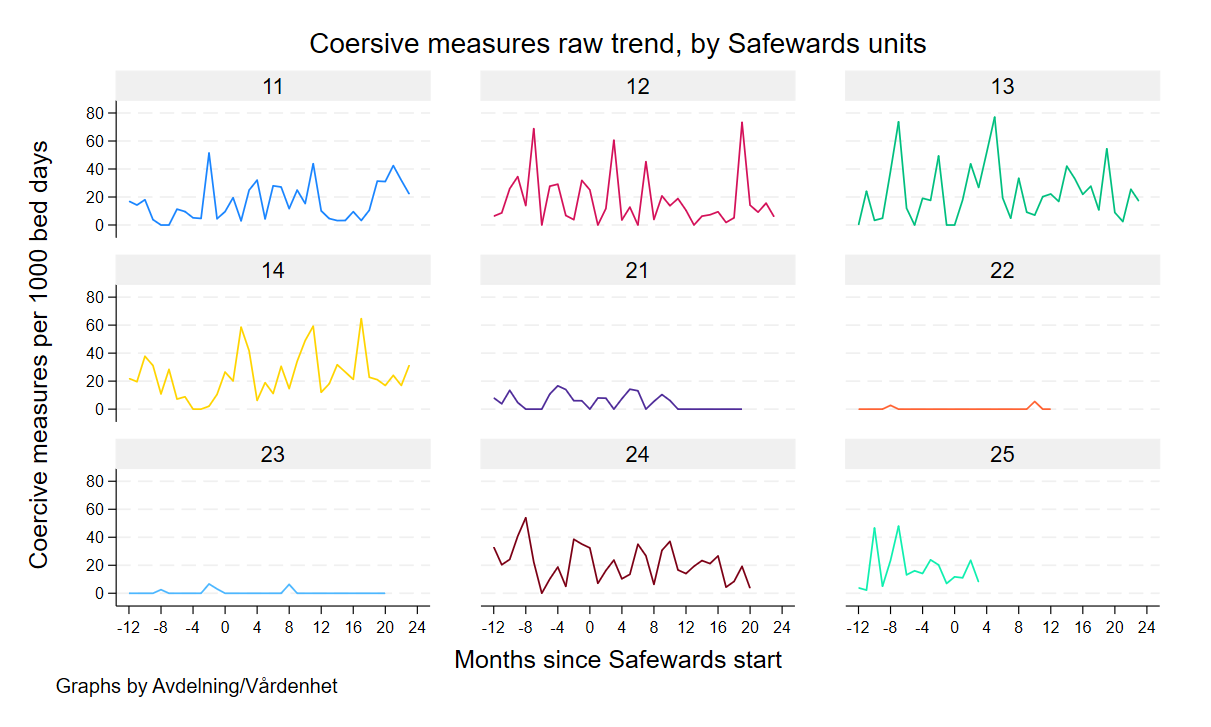
Figure A2. Number of coercive measures per 1000 bed days, by Safewards wards (raw trend)

Note. Ward 11-14 belong to Clinic A, ward 21-25 belong to Clinic B.


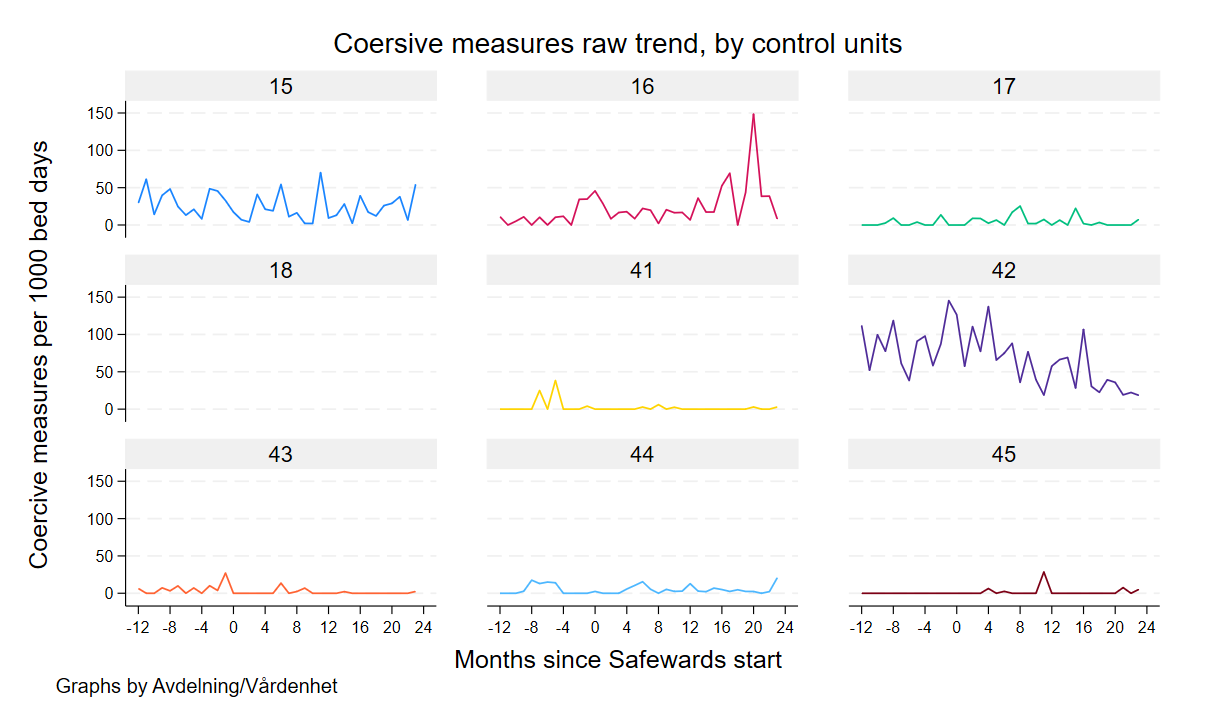
Figure A3. Number of coercive measures per 1000 bed days, by control wards (raw trend)

Note. Ward 15-18 belong to Clinic C, ward 41-45 belong to Clinic D. Ward 18 is excluded from graph due to deviant y-scale.


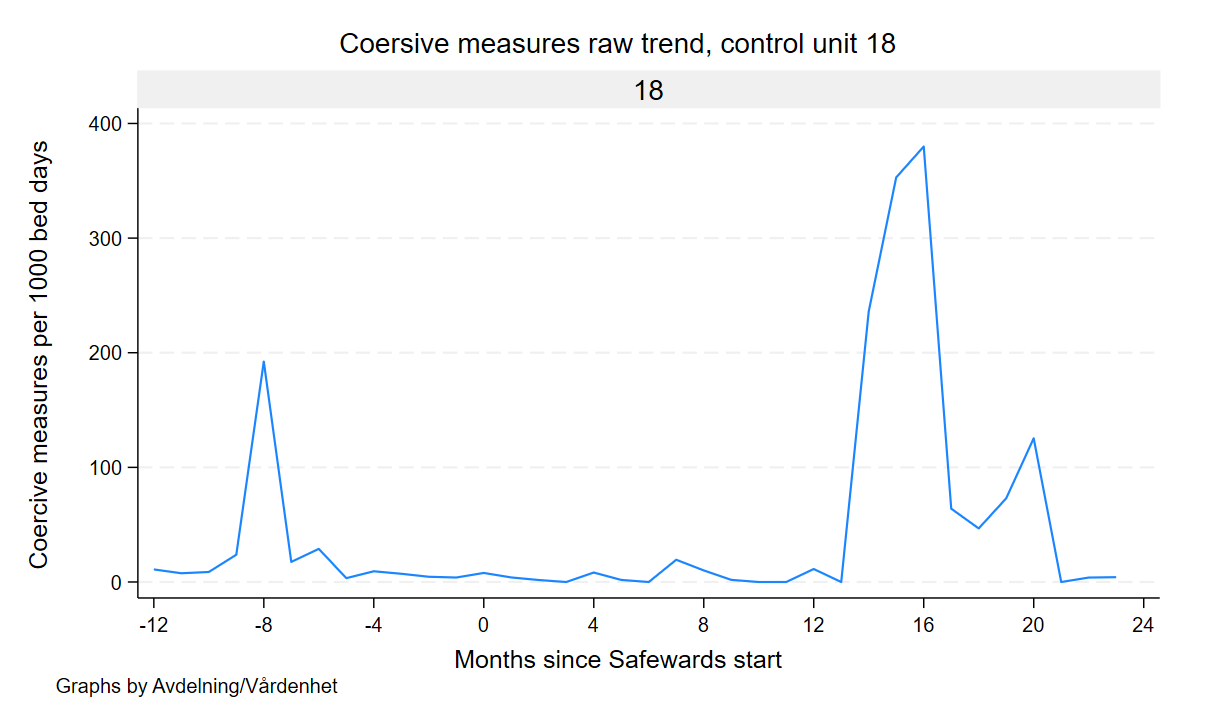
Figure A4. Number of coercive measures per 1000 bed days, in control ward 18 (raw trend)

Note. Control ward 18 presented separately due to deviant y-scale.
